# 4-(2-Amino­phen­yl)-10-oxa-4-aza­tricyclo­[5.2.1.0^2,6^]dec-8-ene-3,5-dione

**DOI:** 10.1107/S160053681100362X

**Published:** 2011-02-09

**Authors:** Jian Li

**Affiliations:** a Department of Chemistry and Chemical Engineering, Weifang University, Weifang 261061, People’s Republic of China

## Abstract

In the title compound, C_14_H_12_N_2_O_3_, the essentially planar pyrrole ring [maximum deviation = 0.037 (4) Å] and the benzene ring form a dihedral angle of 69.5 (2)°. In the crystal, inter­molecular N—H⋯O hydrogen bonds connect mol­ecules into chains along [001]. Additional stabilization is provided by weak inter­molecular C—H⋯O hydrogen bonds.

## Related literature

For the pharmacological applications of 7-oxabicyclo­[2.2.1]hept-5-ene-2,3-dicarb­oxy­lic anhydride and its derivatives, see: Deng & Hu (2007 ▶); Hart *et al.* (2004 ▶). For related structures, see: Li (2010*a* ▶,*b* ▶); Goh *et al.* (2008 ▶).
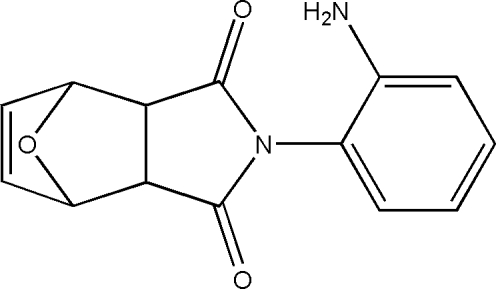


## Experimental

### 

#### Crystal data

C_14_H_12_N_2_O_3_
*M* *_r_* = 256.26
Orthorhombic, 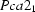

*a* = 10.4457 (11) Å
*b* = 8.8245 (9) Å
*c* = 13.2114 (15) Å
*V* = 1217.8 (2) Å^3^
*Z* = 4
Mo *K*α radiation
μ = 0.10 mm^−1^
*T* = 298 K
0.38 × 0.33 × 0.20 mm

#### Data collection

Bruker SMART CCD diffractometer
Absorption correction: multi-scan (*SADABS*; Bruker, 1997 ▶) *T*_min_ = 0.963, *T*_max_ = 0.980
5021 measured reflections
1131 independent reflections
827 reflections with *I* > 2σ(*I*)
*R*_int_ = 0.061

#### Refinement

*R*[*F* ^2^ > 2σ(*F* ^2^)] = 0.041
*wR*(*F* ^2^) = 0.084
*S* = 1.01
1131 reflections
173 parameters
1 restraint
H-atom parameters constrained
Δρ_max_ = 0.14 e Å^−3^
Δρ_min_ = −0.12 e Å^−3^

### 

Data collection: *SMART* (Bruker, 1997 ▶); cell refinement: *SAINT* (Bruker, 1997 ▶); data reduction: *SAINT*; program(s) used to solve structure: *SHELXS97* (Sheldrick, 2008 ▶); program(s) used to refine structure: *SHELXL97* (Sheldrick, 2008 ▶); molecular graphics: *SHELXTL* (Sheldrick, 2008 ▶) and *PLATON* (Spek, 2009 ▶); software used to prepare material for publication: *SHELXTL*.

## Supplementary Material

Crystal structure: contains datablocks global, I. DOI: 10.1107/S160053681100362X/lh5200sup1.cif

Structure factors: contains datablocks I. DOI: 10.1107/S160053681100362X/lh5200Isup2.hkl

Additional supplementary materials:  crystallographic information; 3D view; checkCIF report

## Figures and Tables

**Table 1:** Hydrogen-bond geometry (Å, °)

*D*—H⋯*A*	*D*—H	H⋯*A*	*D*⋯*A*	*D*—H⋯*A*
N2—H2*B*⋯O2^i^	0.86	2.28	3.131 (5)	174
C3—H3⋯O1^ii^	0.98	2.48	3.232 (5)	133

Symmetry codes: (i) 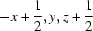; (ii) 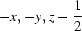.

**Fig. 1.**
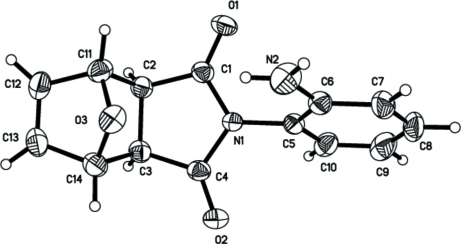
The molecular structure of (I), with displacement ellipsoide drawn at the 30% probability level.

**Fig. 2.**
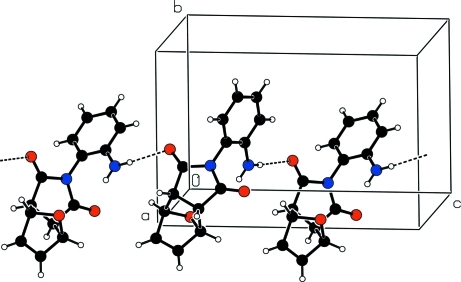
Part of the crystal structure with hydrogen bonds shown as dashed lines.

## supplementary crystallographic information

### Comment

7-Oxa-bicyclo[2,2,1]hept-5-ene-2,3-dicarboxylic anhydride has been widely employed in clinical practice, as it has low toxicity and is relatively easy to synthesize (Deng & Hu, 2007). Its derivatives are pharmacologically active (Hart *et al.*, 2004). In this paper, the structure of the title compound, (I), is reported. The molecular structure of (I) is shown in Fig. 1. The bond lengths and bond angles are as expected and they are comparable to those in similar compounds (Li, 2010*a*,*b*; Goh, *et al.*, 2008). The essentially planar pyrrole ring (maximum deviation = 0.037 (4)Å for atom C2) and the benzene ring form a dihedral angle of 69.5 (2) °. In the crystal, intermolecular N—H···O hydrogen bonds connect molecules into one-dimensional chains along [001]. Additional stabilization is provided by weak intermolecular C—H···O hydrogen bonds.

### Experimental

A mixture of *exo*-7-oxa-bicyclo[2,2,1]hept-5-ene-2,3-dicarboxylic anhydride (0.332 g, 2 mmol) and benzene-1,2-diamine (0.216 g, 2 mmol) in methanol (5 ml) was stirred for 5 h at room temperature, and then refluxed for 1 h. After cooling the precipitate was filtered and dried, the title compound was obtained. The crude product of 20 mg was dissolved in methanol of 10 ml. The solution was filtered to remove impurities, and then the filtrate was left for crystallization at room temperature. The single-crystal suitable for X-ray determination was obtained by evaporation of a methanol solution of the title compound after 5 days.

### Refinement

In the absence of significant anomalous dispersion effects the Friedel pairs were merged. H atoms were initially located in difference maps and then refined in a riding-model approximation with C—H = 0.93–0.98 Å, N—H = 0.86Å and *U*_iso_(H) = 1.2*U*_eq_(C,N).

### Crystal data

**Table:**

C_14_H_12_N_2_O_3_	*F*(000) = 536
*M**_r_* = 256.26	*D*_x_ = 1.398 Mg m^−^^3^
Orthorhombic, *P**c**a*2_1_	Mo *K*α radiation, λ = 0.71073 Å
Hall symbol: P 2c -2ac	Cell parameters from 975 reflections
*a* = 10.4457 (11) Å	θ = 3.1–20.1°
*b* = 8.8245 (9) Å	µ = 0.10 mm^−^^1^
*c* = 13.2114 (15) Å	*T* = 298 K
*V* = 1217.8 (2) Å^3^	Block, pale yellow
*Z* = 4	0.38 × 0.33 × 0.20 mm

### Data collection

**Table:**

Bruker SMART CCD diffractometer	1131 independent reflections
Radiation source: fine-focus sealed tube	827 reflections with *I* > 2σ(*I*)
graphite	*R*_int_ = 0.061
φ and ω scans	θ_max_ = 25.0°, θ_min_ = 2.3°
Absorption correction: multi-scan (*SADABS*; Bruker, 1997)	*h* = −12→11
*T*_min_ = 0.963, *T*_max_ = 0.980	*k* = −10→10
5021 measured reflections	*l* = −15→12

### Refinement

**Table:**

Refinement on *F*^2^	Primary atom site location: structure-invariant direct methods
Least-squares matrix: full	Secondary atom site location: difference Fourier map
*R*[*F*^2^ > 2σ(*F*^2^)] = 0.041	Hydrogen site location: inferred from neighbouring sites
*wR*(*F*^2^) = 0.084	H-atom parameters constrained
*S* = 1.01	*w* = 1/[σ^2^(*F*_o_^2^) + (0.0348*P*)^2^] where *P* = (*F*_o_^2^ + 2*F*_c_^2^)/3
1131 reflections	(Δ/σ)_max_ < 0.001
173 parameters	Δρ_max_ = 0.14 e Å^−^^3^
1 restraint	Δρ_min_ = −0.12 e Å^−^^3^

### Special details

**Table:**

Geometry. All e.s.d.'s (except the e.s.d. in the dihedral angle between two l.s. planes) are estimated using the full covariance matrix. The cell e.s.d.'s are taken into account individually in the estimation of e.s.d.'s in distances, angles and torsion angles; correlations between e.s.d.'s in cell parameters are only used when they are defined by crystal symmetry. An approximate (isotropic) treatment of cell e.s.d.'s is used for estimating e.s.d.'s involving l.s. planes.
Refinement. Refinement of *F*^2^ against ALL reflections. The weighted *R*-factor *wR* and goodness of fit *S* are based on *F*^2^, conventional *R*-factors *R* are based on *F*, with *F* set to zero for negative *F*^2^. The threshold expression of *F*^2^ > σ(*F*^2^) is used only for calculating *R*-factors(gt) *etc*. and is not relevant to the choice of reflections for refinement. *R*-factors based on *F*^2^ are statistically about twice as large as those based on *F*, and *R*- factors based on ALL data will be even larger.

### Fractional atomic coordinates and isotropic or equivalent isotropic displacement parameters (Å^2^)

**Table:**

	*x*	*y*	*z*	*U*_iso_*/*U*_eq_	
N1	0.0406 (3)	0.1444 (3)	0.0863 (2)	0.0401 (7)	
N2	0.2069 (3)	0.1868 (4)	0.2520 (3)	0.0729 (11)	
H2A	0.2110	0.1054	0.2164	0.087*	
H2B	0.2577	0.1992	0.3025	0.087*	
O1	−0.0454 (3)	−0.0189 (3)	0.20351 (18)	0.0680 (9)	
O2	0.1232 (3)	0.2521 (3)	−0.0577 (2)	0.0671 (9)	
O3	0.2418 (2)	−0.1090 (3)	0.03190 (19)	0.0562 (7)	
C1	−0.0025 (4)	0.0040 (4)	0.1192 (3)	0.0467 (9)	
C2	0.0207 (4)	−0.1091 (4)	0.0363 (3)	0.0476 (9)	
H2	−0.0560	−0.1684	0.0201	0.057*	
C3	0.0677 (3)	−0.0159 (4)	−0.0536 (3)	0.0469 (10)	
H3	0.0115	−0.0229	−0.1128	0.056*	
C4	0.0818 (3)	0.1439 (5)	−0.0139 (3)	0.0462 (10)	
C5	0.0363 (4)	0.2805 (4)	0.1474 (3)	0.0442 (9)	
C6	0.1193 (4)	0.2968 (4)	0.2282 (3)	0.0490 (10)	
C7	0.1108 (5)	0.4281 (5)	0.2857 (3)	0.0709 (14)	
H7	0.1657	0.4417	0.3404	0.085*	
C8	0.0224 (6)	0.5379 (5)	0.2631 (4)	0.0818 (16)	
H8	0.0173	0.6247	0.3029	0.098*	
C9	−0.0598 (5)	0.5204 (5)	0.1810 (4)	0.0783 (14)	
H9	−0.1187	0.5958	0.1652	0.094*	
C10	−0.0533 (4)	0.3914 (5)	0.1239 (3)	0.0588 (11)	
H10	−0.1087	0.3780	0.0695	0.071*	
C11	0.1408 (4)	−0.2123 (4)	0.0576 (3)	0.0555 (11)	
H11	0.1455	−0.2562	0.1256	0.067*	
C12	0.1452 (4)	−0.3243 (5)	−0.0286 (3)	0.0640 (12)	
H12	0.1251	−0.4269	−0.0259	0.077*	
C13	0.1827 (4)	−0.2477 (5)	−0.1075 (3)	0.0629 (13)	
H13	0.1946	−0.2846	−0.1728	0.075*	
C14	0.2030 (4)	−0.0868 (4)	−0.0717 (3)	0.0548 (11)	
H14	0.2615	−0.0259	−0.1130	0.066*	

### Atomic displacement parameters (Å^2^)

**Table:**

	*U*^11^	*U*^22^	*U*^33^	*U*^12^	*U*^13^	*U*^23^
N1	0.0467 (17)	0.0387 (18)	0.0349 (15)	−0.0027 (15)	0.0028 (13)	0.0024 (14)
N2	0.076 (3)	0.068 (2)	0.075 (3)	−0.004 (2)	−0.025 (2)	0.004 (2)
O1	0.094 (2)	0.066 (2)	0.0437 (16)	−0.0215 (16)	0.0186 (16)	0.0025 (14)
O2	0.093 (2)	0.0526 (19)	0.0561 (17)	−0.0006 (16)	0.0217 (16)	0.0131 (14)
O3	0.0502 (17)	0.0539 (16)	0.0644 (17)	−0.0025 (15)	−0.0137 (14)	−0.0007 (13)
C1	0.051 (2)	0.049 (2)	0.040 (2)	−0.008 (2)	−0.0003 (18)	−0.0011 (17)
C2	0.052 (2)	0.048 (2)	0.0433 (19)	−0.009 (2)	0.0000 (18)	−0.0039 (19)
C3	0.051 (2)	0.051 (2)	0.038 (2)	0.005 (2)	−0.0063 (17)	0.0006 (18)
C4	0.047 (2)	0.050 (3)	0.042 (2)	0.006 (2)	0.0011 (18)	0.009 (2)
C5	0.046 (2)	0.042 (2)	0.045 (2)	0.002 (2)	0.0095 (18)	0.0005 (18)
C6	0.053 (3)	0.049 (2)	0.045 (2)	−0.004 (2)	0.0000 (19)	−0.0003 (19)
C7	0.086 (4)	0.065 (3)	0.062 (3)	−0.027 (3)	0.014 (2)	−0.019 (2)
C8	0.095 (4)	0.053 (3)	0.097 (4)	−0.012 (3)	0.049 (3)	−0.028 (3)
C9	0.068 (4)	0.051 (3)	0.115 (4)	0.016 (3)	0.027 (3)	0.003 (3)
C10	0.055 (3)	0.047 (2)	0.075 (3)	0.003 (2)	0.008 (2)	0.004 (2)
C11	0.067 (3)	0.050 (2)	0.050 (2)	−0.003 (2)	−0.006 (2)	0.0055 (18)
C12	0.076 (3)	0.046 (3)	0.070 (3)	0.005 (2)	0.001 (2)	−0.008 (2)
C13	0.069 (3)	0.061 (3)	0.059 (3)	0.014 (2)	0.002 (2)	−0.013 (2)
C14	0.056 (3)	0.061 (3)	0.047 (2)	0.006 (2)	0.0046 (19)	0.003 (2)

### Geometric parameters (Å, °)

**Table:**

N1—C1	1.388 (4)	C5—C6	1.383 (5)
N1—C4	1.392 (4)	C5—C10	1.390 (5)
N1—C5	1.447 (4)	C6—C7	1.388 (5)
N2—C6	1.370 (5)	C7—C8	1.372 (7)
N2—H2A	0.8600	C7—H7	0.9300
N2—H2B	0.8600	C8—C9	1.392 (7)
O1—C1	1.217 (4)	C8—H8	0.9300
O2—C4	1.198 (4)	C9—C10	1.367 (6)
O3—C11	1.434 (4)	C9—H9	0.9300
O3—C14	1.440 (4)	C10—H10	0.9300
C1—C2	1.502 (5)	C11—C12	1.508 (5)
C2—C3	1.525 (5)	C11—H11	0.9800
C2—C11	1.576 (5)	C12—C13	1.303 (6)
C2—H2	0.9800	C12—H12	0.9300
C3—C4	1.512 (5)	C13—C14	1.512 (5)
C3—C14	1.564 (5)	C13—H13	0.9300
C3—H3	0.9800	C14—H14	0.9800
			
C1—N1—C4	113.3 (3)	C8—C7—C6	120.9 (5)
C1—N1—C5	123.8 (3)	C8—C7—H7	119.5
C4—N1—C5	122.9 (3)	C6—C7—H7	119.5
C6—N2—H2A	120.0	C7—C8—C9	120.4 (4)
C6—N2—H2B	120.0	C7—C8—H8	119.8
H2A—N2—H2B	120.0	C9—C8—H8	119.8
C11—O3—C14	96.0 (3)	C10—C9—C8	119.5 (4)
O1—C1—N1	123.6 (3)	C10—C9—H9	120.3
O1—C1—C2	128.1 (4)	C8—C9—H9	120.3
N1—C1—C2	108.2 (3)	C9—C10—C5	119.8 (4)
C1—C2—C3	105.2 (3)	C9—C10—H10	120.1
C1—C2—C11	112.4 (3)	C5—C10—H10	120.1
C3—C2—C11	101.2 (3)	O3—C11—C12	102.5 (3)
C1—C2—H2	112.4	O3—C11—C2	100.2 (3)
C3—C2—H2	112.4	C12—C11—C2	105.5 (3)
C11—C2—H2	112.4	O3—C11—H11	115.6
C4—C3—C2	105.3 (3)	C12—C11—H11	115.6
C4—C3—C14	109.8 (3)	C2—C11—H11	115.6
C2—C3—C14	101.2 (3)	C13—C12—C11	105.9 (4)
C4—C3—H3	113.2	C13—C12—H12	127.1
C2—C3—H3	113.2	C11—C12—H12	127.1
C14—C3—H3	113.2	C12—C13—C14	106.1 (4)
O2—C4—N1	124.7 (4)	C12—C13—H13	126.9
O2—C4—C3	127.7 (4)	C14—C13—H13	126.9
N1—C4—C3	107.6 (3)	O3—C14—C13	102.1 (3)
C6—C5—C10	121.4 (3)	O3—C14—C3	99.4 (3)
C6—C5—N1	119.9 (3)	C13—C14—C3	107.3 (3)
C10—C5—N1	118.7 (3)	O3—C14—H14	115.4
N2—C6—C5	121.4 (3)	C13—C14—H14	115.4
N2—C6—C7	120.6 (4)	C3—C14—H14	115.4
C5—C6—C7	118.0 (4)		
			
C4—N1—C1—O1	−178.2 (4)	C10—C5—C6—C7	0.0 (5)
C5—N1—C1—O1	−1.7 (5)	N1—C5—C6—C7	−179.1 (3)
C4—N1—C1—C2	4.9 (4)	N2—C6—C7—C8	−179.2 (4)
C5—N1—C1—C2	−178.6 (3)	C5—C6—C7—C8	0.1 (6)
O1—C1—C2—C3	176.8 (4)	C6—C7—C8—C9	−0.7 (7)
N1—C1—C2—C3	−6.4 (4)	C7—C8—C9—C10	1.1 (7)
O1—C1—C2—C11	−73.9 (5)	C8—C9—C10—C5	−0.9 (6)
N1—C1—C2—C11	102.8 (3)	C6—C5—C10—C9	0.4 (6)
C1—C2—C3—C4	5.6 (4)	N1—C5—C10—C9	179.5 (3)
C11—C2—C3—C4	−111.6 (3)	C14—O3—C11—C12	48.6 (3)
C1—C2—C3—C14	119.9 (3)	C14—O3—C11—C2	−59.9 (3)
C11—C2—C3—C14	2.7 (3)	C1—C2—C11—O3	−77.4 (3)
C1—N1—C4—O2	−179.7 (4)	C3—C2—C11—O3	34.3 (3)
C5—N1—C4—O2	3.7 (6)	C1—C2—C11—C12	176.5 (3)
C1—N1—C4—C3	−1.1 (4)	C3—C2—C11—C12	−71.8 (4)
C5—N1—C4—C3	−177.7 (3)	O3—C11—C12—C13	−31.7 (4)
C2—C3—C4—O2	175.5 (4)	C2—C11—C12—C13	72.7 (4)
C14—C3—C4—O2	67.3 (5)	C11—C12—C13—C14	0.3 (5)
C2—C3—C4—N1	−3.0 (4)	C11—O3—C14—C13	−48.3 (3)
C14—C3—C4—N1	−111.2 (3)	C11—O3—C14—C3	61.8 (3)
C1—N1—C5—C6	72.1 (4)	C12—C13—C14—O3	31.1 (4)
C4—N1—C5—C6	−111.7 (4)	C12—C13—C14—C3	−72.9 (4)
C1—N1—C5—C10	−107.0 (4)	C4—C3—C14—O3	72.1 (3)
C4—N1—C5—C10	69.2 (5)	C2—C3—C14—O3	−38.9 (3)
C10—C5—C6—N2	179.4 (3)	C4—C3—C14—C13	178.0 (3)
N1—C5—C6—N2	0.3 (5)	C2—C3—C14—C13	67.0 (3)

### Hydrogen-bond geometry (Å, °)

**Table:**

*D*—H···*A*	*D*—H	H···*A*	*D*···*A*	*D*—H···*A*
N2—H2B···O2^i^	0.86	2.28	3.131 (5)	174
C3—H3···O1^ii^	0.98	2.48	3.232 (5)	133

Symmetry codes: (i) −*x*+1/2, *y*, *z*+1/2; (ii) −*x*, −*y*, *z*−1/2.
